# Gender Disparities in Pandemic-Related Strains, Digital Coping Strategies, and Protective Mechanisms Among Rural-to-Urban Migrant Working Adolescents in China

**DOI:** 10.3390/bs15010073

**Published:** 2025-01-16

**Authors:** Xinge Jia, Hua Zhong, Qian Wang, Qiaobing Wu

**Affiliations:** 1 Department of Sociology, 4/F Sino Building, The Chinese University of Hong Kong, Shatin NT, Hong Kong 999077, China; xinge.jia@link.cuhk.edu.hk; 2 Department of Psychology, The Chinese University of Hong Kong, Hong Kong 999077, China; qianwang@cuhk.edu.hk; 3 Department of Applied Social Sciences, The Hong Kong Polytechnic University, Hong Kong 999077, China; qiaobing.wu@polyu.edu.hk

**Keywords:** migrant worker, Chinese adolescents, general strain theory, internet use

## Abstract

The COVID-19 pandemic placed significant strains on daily life, particularly affecting vulnerable groups such as rural-to-urban young migrant workers. Based on General Strain Theory (GST), these pandemic-related strains lead to delinquent copings, including excessive Internet use. However, the association between pandemic-related challenges faced by migrant youth and their digital copings has yet to be investigated. GST also posits that some conditioning factors, such as conventional beliefs, internal resilience and life satisfaction, might serve as protective factors, which can help to alleviate the disruptive consequences of the pandemic-related strains. Utilizing the fourth sweep of International Self-Report Delinquency Survey (ISRD4) in China comprising 769 working migrant adolescents aged 16 to 19, who did not attend high school, the present study examines variations in pandemic-related strains, frequent use of the Internet for gaming and social media, and their associations. In addition, this study investigates the moderating effect of three protective factors: conventional beliefs, internal resilience and life satisfaction. Results indicated that economic strain, information strain and health-related strain significantly influenced digital coping strategies, with notable gender differences. Conventional beliefs served as a significant moderator for males, while life satisfaction played a more significant moderating role for females. Relevant policy implications are then discussed.

## 1. Introduction

The sudden onset of the Covid-19 pandemic and the implementation of restrictive policies have dramatically altered people’s daily routines. Worldwide studies showed that the changes brought about by pandemic significantly elevated stress-related experiences to unprecedented levels (Gloster et al., 2020; C. Wang et al., 2020). Rising infection rates, death tolls, business closures, travel restrictions, and the shift to working from home have all contributed to increased stress (K. L. Newman et al., 2022).

Exposure to multiple pandemic-related strains has an adverse impact on people’s mental health (Sebastião et al., 2023), increasing anxiety, depression and loneliness (Elmer et al., 2020; J. Gao et al., 2020). Beyond the virus itself, financial insecurity, occupational difficulties, life instability and limited resources are all pandemic-driven stressors that harm mental health (Pearlin & Bierman, 2013). However, these pandemic-related strains do not impact all social groups equally. Some people are more vulnerable and face greater difficulty in recovering from such adversities.

Adolescents are among the most vulnerable groups during health crises. Due to the ongoing development in brain, behavioral and cognitive systems, adolescence is a period of high vulnerability (Steinberg, 2005). Not surprisingly, most studies in China observed increased strains and deterioration in adolescents’ mental health during the pandemic (Cao et al., 2022; S. Wu et al., 2021). A survey of 687 people in Wuhan found that students were among the most vulnerable to depression, anxiety and stress during the lockdown (Du et al., 2020). In addition, a survey of 8079 Chinese adolescents aged 12 to 18 from 21 provinces and autonomous regions revealed high prevalence rates of depression (43.7%) and anxiety (37.4%) during the pandemic (Zhou et al., 2020).

Migrant workers are also a vulnerable group under the pandemic, especially in China. Since the 1980s, industrialization and urbanization have driven a large migration of the rural population to urban areas for better job opportunities and higher living standards (P. Li & Li, 2007). However, due to the Chinese hukou system, migrant workers without urban hukou cannot access the same social welfare and services as urban residents, such as housing subsidies, public schools for children, healthcare, and pension insurance (Chan, 2010). The majority of migrant workers are excluded from certain urban industries and mainly work in informal, low-skilled jobs (Wong et al., 2007). The pandemic has exacerbated their vulnerability in workplace. With sudden economic shocks and reduced demand for informal jobs, those without job security are more likely to be laid off or face pay cuts (Tang & Li, 2021). In addition, limited medical insurance means fewer resources to protect themselves from infection and incur higher costs if they do get sick. These social inequalities are exacerbated by the pandemic.

In China, there exists a group of adolescents who have already entered the workforce, as people over 16 are legally allowed to work (State Council of the People’s Republic of China, 2002). After nine years of compulsory education, students attend high schools based on their entrance exam scores (*Zhongkao*) (X. Wang et al., 2020). Those who do not achieve the required grades often drop out of school and begin to work, mostly from rural areas (Lu & Lai, 2009). Given their dual vulnerability as both adolescents and migrant workers, migrant working adolescents are likely to experience more pandemic-related strains. Under pressure, people may turn to delinquent and criminal behaviors to relieve strains (Agnew, 1992). Actually, the increasing influx of migrants has been attributed as a factor in the rise in crime rates (Lo & Jiang, 2006). Migrant adolescents are commonly reported as a disproportionately high group with delinquent behaviors (Y. Gao & Wong, 2018), with this phenomenon being especially pronounced among male migrant adolescents (Fernández-Pacheco Alises et al., 2022). Therefore, it is worth studying the pandemic-related strains on this unique group and examining whether they use delinquent coping strategies for strain relief, while also paying attention to gender disparities.

Until now, existing studies have focused on the pandemic’s impact on individual vulnerable groups, such as adult migrant workers or school adolescents. However, research on migrant working adolescents, who possess vulnerabilities of both groups, is scarce. Moreover, no studies have specifically explored the strains faced by this group, including gender differences. Whether this group is likely to engage in delinquent behaviors and contribute to social instability remains understudied. Based on these gaps, the current study aims to examine, with a particular emphasis on gender differences, diverse pandemic-related strains experienced by both male and female migrant working adolescents, the associations between strains and different delinquent coping strategies, and possible protective factors for these vulnerable youth.

## 2. General Strain Theory and Pandemic-Related Strains

General Strain Theory (GST) offers a useful framework to understand the potential mechanism of pandemic-related strains and delinquent coping strategies. Traditional strain theories describe strain as the contradiction between culturally accepted goals and institutional means (Merton, 1938). Drawing from this framework, Agnew (2006) expanded this theory and proposed GST by defining strain as events or conditions disliked by individuals experiencing them. Instead of only focusing on one type of negative relationship, Agnew (1992) identified three major sources of strains: failure to achieve positively valued goals, loss of positively valued stimuli, and presentation of negative or noxious stimuli. In the absence of adequate coping strategies, people may use delinquent behaviors to escape from these negative conditions.

The outbreak of the pandemic and restrictive policies undoubtedly created adverse conditions, leading to various strains. This study specifically focused on four sources of strains. Firstly, during the global economic crisis, financial issues worsened for already vulnerable groups (Brodeur et al., 2021). Scholars like Bapuji et al. (2020) noted that workers in developing countries’ informal sectors were greatly impacted. At the initial stage, rising unemployment forced six percent of China’s employees out of the labor market, and at least 50 million migrant workers exited the urban labor market (He et al., 2022). Unlike urban workers with unemployment insurance, migrant workers struggled to recover from financial strain without social assistance. With limited work experience and savings, migrant working adolescents face even greater economic challenges. Secondly, the pandemic also brought about an exceptional ‘infodemic ’due to the rapid spread of false or misleading information and conflicting messages on social media (Eysenbach, 2002). This unreliable information negatively impacted people’s health behaviors and eroded public trust in the health care system (Nsoesie & Oladeji, 2020). With about two-thirds of China’s population using social media, the infodemic was severer. Almost 87% of users encountered misinformation during the pandemic (Jiang, 2020). Similarly, health-related strain is one major type of pandemic-related strains. The widespread virus caused fear of infection (Taylor et al., 2020), worry about the shortage supply of pharmaceutical products and food, and concerns about the capability of the local medical system (Socal et al., 2021). Studies showed that during the pandemic, adolescents were more concerned about the health of themselves, their family members, and friends (Styck et al., 2021; Villaume et al., 2021). Finally, the pandemic altered work–family dynamics. Increasing demands on families led to greater conflicts between work and family, which caused more stress for parents (Schnettler et al., 2024). This increased stress led to more frequent disputes among family members. As they spent more time together, many parents reported more conflicts with their children during the pandemic (Gadermann et al., 2021). These conflicts likely contributed to greater family relationship strain for adolescents.

GST has been considered one theory to explain gender differences in crime and deviance (Broidy & Agnew, 1997). Due to traditional gender roles and hierarchies, males are often expected to be the family breadwinners and value highly on material success. Consequently, they are more likely to suffer from economic strain if they fail to achieve their monetary goal. In China, this labor division between men and women is more distinguished. During the pandemic, studies showed males were more likely to experience financial hardship. For example, a UK study found that men faced more economic difficulties while women, earning less and working shorter hours, were somewhat protected from layoffs (Witteveen, 2020). Similarly, a study in India found that men were more likely to lose jobs during the pandemic-induced economic recession (Hossain, 2021). Instead, women are more concerned with maintaining close relationships with intimate individuals. Hence, they experience more interpersonal strains. In addition, females are more susceptible to health-related strains. A study on Italy workers showed that women were more likely than men to be extremely worried and afraid of this new virus (Giudice et al., 2022). A review article also concluded that females perceive the virus as a greater threat to people’s health than males (Metin et al., 2022). However, it remains unclear whether similar gender differences in strains exist among Chinese migrant working adolescents.

## 3. Digital Copings Under the Pandemic

Based on GST, in the absence of adequate suitable copings, people may turn to delinquent coping strategies to relieve strains and relevant negative emotions (Agnew, 1992). Delinquent behaviors include illegal or problematic behaviors, such as Internet Addiction (IA), that harm adolescents’ safety, physical health, and mental well-being (Kim et al., 2013; Liu et al., 2018). Immersing oneself in the Internet can serve as a coping strategy to alleviate perceived strain. Previous studies affirmed the link between strain and excessive Internet use (Gong et al., 2021). In this study, we consider delinquent behaviors as excessive use of the Internet that adversely impacts adolescents.

In the context with many restrictive policies, various forms of social isolation made people highly dependent on digital devices for work, study and socialization. Online activities like gaming and social networking can reduce the stress and discomfort from physical isolation while maintaining social connection and providing entertainment (Wegmann et al., 2021). Therefore, Internet use can be a popular and practical coping strategy to relieve pandemic-associated strains. A study in the Middle East region indicated that 53.2% of participants spent 6 or more hours online daily, with 31.5% spending over 8 h online during the lockdown (Alheneidi et al., 2021). Accordingly, this raises concerns about whether the increased Internet use leads to more digital addiction among adolescents. In China, the average daily Internet use among adolescents has risen significantly compared to the pre-pandemic period, which may increase the risk of IA (Duan et al., 2020; Lin, 2020). Another study found that only 55.52% maintained the suitable Internet behavior during the pandemic, while 5.28% developed IA (Q. Wu et al., 2022). However, the association between pandemic-related strains and excessive Internet use among migrant working adolescents in China is not well studied yet.

Internet game disorder and social media addiction are screen activities that can lead to IA. Gender differences are evident in these behaviors. Usually, boys use the Internet more frequently and have a greater interest in playing online games, such as massively multiplayer online role-playing games, while girls spend more time on social networks (Dufour et al., 2016; Leonhardt & Overå, 2021). According to Hill and Lynch’s (1983) gender intensification hypothesis, adolescents face growing social pressures from parents, peers, teachers, and media to conform to culturally defined gender roles, leading to more distinct gender role identities into adulthood. This hypothesis provides an explanation for the growing gender differences in screen activities during adolescence. Given that male aggressiveness is more socially accepted, many games are designed with violent and adventurous elements that appeal more to male adolescents (Barua & Barua, 2012). In contrast, female adolescents are more inclined to create their identities through new practices related to puberty and are less interested in childhood activities like online video games (Leonhardt & Overå, 2021). Compared to boys, girls focus more on interpersonal relationships and appearance, and tend to be more obliging and obedient in their interactions. Hence, they find social interactions on social media more engaging.

Considering motivation for using media, the Uses and Gratifications theory (UGT) is an alternative perspective to explain gender differences in digital use (Katz et al., 1973). UGT offers a valuable theoretical framework by conceptualizing media selection and use as a goal-driven process to fulfill people’s specific needs, which can be categorized into cognitive, affective, integrative, social integrative, and escapist dimensions (Katz, 1974). Individuals’ media choices, participation forms, and the resulting benefits are shaped by their conscious objectives and intentions, which are influenced by their cultural and social environments (Katz et al., 1973). UGT is useful in exploring how gender, as a social structure, can translate into different motivations. Studies suggest that men and women pursue different gratifications through various digital activities, which influence their media selection and use patterns. For example, one study revealed that male players prioritize game-related gratifications more highly than women (Sherry et al., 2012). This highlights the importance of online games for males. Consistent with pre-pandemic studies, studies under the pandemic also showed that males play online games more frequently and are at a higher risk of game disorder (L. Li et al., 2023; Stevens et al., 2021). In contrast, other studies found that females are more motivated to use social media for relieving stress and fostering social interactions (P. Li & Zhuo, 2023). Strains are more significant predictors for social media addiction among females (Tu et al., 2023). Despite these insights, gender differences in digital coping strategies for various pandemic-related strains remain unexplored, particularly among migrant working adolescents. Understanding their gender-specific digital coping behaviors can inform targeted interventions and support strategies.

## 4. Protective Factors

Agnew (1992, 2006) pointed out that delinquency is one possible response to strain, and is particularly likely to occur when (1) non-delinquent behaviors are highly constrained (e.g., lack of legal problem-solving skills and social support); (2) the cost of delinquent behaviors is low (it’s hard to be detected and punished) and (3) there is a high propensity for delinquent behaviors (e.g., with low self-control). In other words, strains and accompanying negative emotion do not necessarily cause delinquency. Multiple factors can condition the impact of strains (Agnew, 2013).

Agnew (1992) highlighted the conditioning role of conventional beliefs in impacting responses to strains. Consistent with Hirschi’s social bond theory (1969), conventional beliefs are key components of self-controls, which are important factors in crime prevention. These beliefs reflect individuals’ acknowledgment of social norms’ moral legitimacy. Those who accept crime and delinquency are less likely to reject deviant behaviors. GST posits that differences in conventional beliefs lead to varied copings to strains, where weaker moral constraints increase the likelihood of delinquent behaviors. A study confirmed that the impact of strains on delinquency intensified when conventional beliefs were weak (Mazerolle & Maahs, 2000). Conversely, stronger conventional beliefs foster self-control and goal commitment, reducing the appeal of delinquency. Empirical studies corroborated the negative relationship between belief in conventional norms and delinquent behaviors (Junger-Tas, 1992; Wiatrowski & Swatko, 1979). Moreover, there are gender differences in beliefs towards crime and delinquency through varying socialization experiences (Friedman & Rosenbaum, 1988). Men generally develop individuality, while women focus on maintaining relationships (Gilligan, 1993). This relational focus can promote women’s awareness of the moral binding nature of social norms (Chapple et al., 2005). However, empirical findings on gender difference in attitudes towards crimes yield inconsistent results. Some studies found that compared with men, women tend to judge crimes such as assault and burglary (Rauma, 1991), and overall crimes (Borg & Hermann, 2023) more harshly, while other studies reported no significant gender difference (G. R. Newman & Wolfgang, 2017; Wolfgang, 1985).

Furthermore, Agnew et al. (2002) identified personality traits as important conditioning factors, which can significantly impact emotional reactions, coping strategies, perception of criminal costs, and criminal tendencies. Positive emotionality is a core personality trait in GST literature. People with higher levels of well-being, social potency, achievement and social closeness are less likely to respond to strains with criminal behaviors (Waller et al., 1991). Resilient individuals tend to exhibit higher levels of positive emotionality (Block & Kremen, 1996; Fredrickson & Levenson, 1998). This may explain why some adolescents maintain mental well-being and avoid problematic behaviors despite adversity. Their positive personal traits can disrupt the link between risk factors and negative outcomes (Fergus & Zimmerman, 2005). In short, internal resilience helps youth adapt positively to adversity and mitigate the negative effect of risks. In this study, we consider internal resilience as a positive personality trait that helps individuals accept themselves, adapt to life changes, and cope with challenges. Greater resilience was found to reduce mental health problems and problematic behaviors among adolescents (Ali et al., 2010; Q. Wu et al., 2018), and was related to better educational outcomes among Chinese migrant children (Q. Wu et al., 2014). Additionally, a systematic review further confirmed the negative relationship between resilience and problematic Internet use (Hidalgo-Fuentes et al., 2023). However, findings about the impact of gender on resilience are mixed. Some studies found that males tended to have greater resilience (Erdogan et al., 2015; Hou et al., 2020), while other studies suggested no gender difference (Jillani et al., 2023; Kobayasi et al., 2018).

In addition to internal resilience, life satisfaction, a common measure of well-being, acts as a protective factor that deters adolescents from engaging in criminal activities. Drawing from positive psychology, studies began to identify the relationship between well-being and delinquent behaviors (Martin et al., 2008). For example, a study with 1201 middle and high school students revealed those with higher levels of life satisfaction were less likely to have delinquent and aggressive behaviors, even under pressure (Suldo & Huebner, 2004). Furthermore, high life satisfaction is an effective protective factor against problematic Internet use (Dhir et al., 2015; Kim et al., 2009). However, the role of life satisfaction in the relationship between different pandemic-related strains and Internet use is unclear. Moreover, girls typically report lower levels of life satisfaction than boys (Inchley et al., 2016). This gender disparity is particularly pronounced in societies like China, where gender inequality limits females’ access to privileges and opportunities. Hence, it is important to understand the protective role of life satisfaction against delinquency across genders in China. This can guide gender-specific interventions to enhance life satisfaction and reduce delinquency.

## 5. Study Purpose and Research Questions

Existing studies have extensively explored pandemic-related strains and coping strategies across various populations. However, few studies related these concerns with migrant working adolescents. As a unique vulnerable group, the pandemic-related strains they experienced and the coping strategies they used warrant attention. In addition, it remains unclear whether gender differences in strains and digital coping strategies observed in other populations also apply to this group. The inconsistent results of gender differences in protective factors further highlight the need to explore their impacts on migrant working adolescents. Building on these gaps, this study aims to investigate the impact of the pandemic on this group, with a focus on gender difference. The research questions are as follows: for migrant working adolescents in China,

Research question 1: What was the impact of Covid-19 on their stress level, and were there any gender differences?

Research question 2: What was the relationship between various strain types and different digital copings? Did the relationship vary by gender?

Research question 3: What was the impact of protective factors, i.e., conventional beliefs, internal resilience, life satisfaction, on the relationship between strains and digital copings? Did the impact vary by gender?

We firstly hypothesized that males experience higher levels of economic strain, while females face more information, health-related and family relationship strains. Males are expected to play online games more frequently, whereas females are more likely to use social media. In addition, we hypothesized that pandemic-related strains affect digital coping strategies, with males more inclined to use gaming and females preferring social media. Finally, we proposed that conventional beliefs, internal resilience, and life satisfaction moderate the relationship between strains and digital copings, with different effects by gender.

## 6. Materials and Methods

### 6.1. Participants

Data for this study were drawn from the fourth sweep of the International Self-Report Delinquency Study (ISRD4) in China. This self-reported survey was administered between September 2021 and July 2022. The ISRD, an international and collaborative project, investigated adolescent victimization and delinquency across about 35 countries from 2021 to 2023. It employed a standardized set of questions alongside a country-specific module developed by local teams. In China, this module focused on the impact of Covid-19 on delinquent behaviors among vulnerable adolescents.

Participants in this study were working migrant adolescents aged 16–19. As they had left school, this vulnerable group was hard to reach, which partly explains why prior studies focused more on migrant children at school. With these important concerns, an Internet survey was completed in fall 2022. Compared to conventional face-to-face surveys, Internet surveys are able to rapidly collect data with a large sample size. The questionnaire was translated from English to Chinese and participation was entirely voluntary. The research ethics were approved by the authors’ university. Considering regional economic differences, Shenzhen and Changsha were selected as two cities for the survey. Shenzhen has one of the largest migrant populations in China, with the number continually increasing. In 2019, among the 13.44 million residents, only 4.95 million have Shenzhen urban hukou (residency) (Cai & Guo, 2020). Changsha, the capital of Hunan province and a key city in Central China, is less developed than Shenzhen but also has many migrant workers. It has a permanent population of over 8 million. In one district of Changsha in 2023, 38,000 out of the 822,000 people with registered hukou were classified as having rural hukou. (Government of Yuhua District, Changsha, Population, 2023).

Finally, 1391 participants aged 16 to 19 returned useful questionnaire, with 876 from Shenzhen and 515 from Changsha. Given that different family backgrounds, whether urban-native or migrant, may influence youth’s Internet use (Chang et al., 2016), and aligning with the study’s purpose, only migrant working students were considered. Urban native and urban-to-urban migrant adolescents were excluded. The final sample has 769 migrant working adolescents, with 446 from Shenzhen and 323 from Changsha.

### 6.2. Measurement

#### 6.2.1. Dependent Variables

Frequent Internet Use. The current study used changes in adolescents’ Internet use compared to the pre-pandemic period as the dependent variable. The survey included the question, “Compared to preCovid-19, did the average amount of time you spend per day on the following online activities change during the last 12 months?” followed by four questions related to Internet use. For this study, two questions about Internet use for leisure activities were selected: (1) “To play games”, (2) “To use social media (TikTok, Wechat, QQ and Weibo)”. Response options were: 1 = more often, 2 = as often as in other times, 3 = Less often, 4 = No change because I seldom do these things. For each item, response one was coded as 1 (frequent Internet use) and the other responses were coded as 0. In sum, we have two dummy dependent variables.

#### 6.2.2. Independent Variables

Pandemic-related strain. Pandemic-related strain consists of economic strain, information strain, health-related strain, and family relationship strain. Economic strain was measured by summing the scores of four items. Two items assessed the participant’s economic condition, asking whether they became unemployed or had significantly lower income in the past 12 months. The other two items evaluated their parents’ economic condition, asking if the respondent’s father or mother became unemployed in the past 12 months. Responses “Yes” were coded as 1 and “No” as 0. Then the scores across items were summed (Cronbach’s alpha = 0.64). Higher values are associated with higher levels of economic strain. Information strain was assessed by three items that asked respondents the extent to which they felt bothered by an overload of COVID-19-related news, false or misleading information, and inconsistent information. Each item was rated on a 5-point Likert scale (1 = not at all, 5 = very much). Higher values represent higher levels of information strain (alpha = 0.81). Health-related strain was measured using a sum of nine items that assessed respondents’ worries about infection and the shortage of disinfectant supplies and medicines, etc. Respondents rated each item on a 5-point Likert scale (1 = not worried at all, 5 = very worried). Higher scores correspond to higher levels of health-related strain (alpha = 0.95). Family relationship strain was assessed by two items assessing conflicts between parents in the past 12 months, “whether your parents got into physical fights”; and “whether your parents had very heated arguments with each other” (0 = no, 1 = yes). Scores for each item were summed. Higher scores represent higher levels of family relationship strain (alpha = 0.64).

#### 6.2.3. Moderating Variables

Conventional beliefs. Questions about opinions on violence were used to measure conventional beliefs. Respondents were presented with 7 scenarios such as “Sharing online an embarrassing photo or video of someone that he or she did not want others to see” and “Hitting another person without causing injury”, and asked to rate the extent to which they personally see these acts as violence. Each item consists of a 4-point scale (1 = Not at all, 4 = Absolutely). Scores for each item were summed, with higher scores indicating stronger conventional beliefs (alpha = 0.86).

Internal resilience. We employed a 10-item resilience scale developed by Campbell-Sills and Stein (2007). This scale assesses respondents’ capacity to manage diverse challenges, including change, personal issues, stress, failure, illness, and painful feelings. Each item was rated on a 5-point Likert scale (1 = not true at all, 5 = true nearly all the time). Higher values indicate higher levels of internal resilience (alpha = 0.96). To address the low frequency in some score categories, we reclassified the variable by grouping scores into four ranges: 20 and below, over 20 to 30, over 30 to 40, and 41 and above.

Life Satisfaction. To measure life satisfaction, we used the question, “Think back over the last six months: Would you say that most of the time you have been happy?”. This question directly measures happiness, which can be viewed as an individual’s subjective sense of overall life enjoyment, also known as “life satisfaction” (Veenhoven, 2017). Respondents rated it on a 6-point scale (1 = Very happy, 6 = Very unhappy). The responses were reversely coded so that higher values indicate higher levels of life satisfaction.

We also adjusted for gender (1 = male, 0 = female), city (1 = Shenzhen, 0 = Changsha), and family intactness (1 = mainly live with parents, 0 = otherwise).

#### 6.2.4. Statistical Analysis

The analytic strategy for this study was conducted in the following steps. Firstly, descriptive statistics for the study variables were reported. The *t*-test/chi-square test was then conducted between males and females for the dependent variable and each predictor to examine different online behaviors and pandemic-related strains. Next, a logistic regression model was used to test GST. This model was first applied to the entire sample and then separately for each gender. Finally, to identify the moderating effect of conventional beliefs, internal resilience and life satisfaction, interaction terms with the four pandemic-related strains variables were added to the model. All statistical models were fitted using STATA version 17.0. All continuous predictors were standardized for consistent parameter interpretation.

## 7. Results

### 7.1. Group Variations in Pandemic-Related Strain and Frequent Internet Use

Table 1 indicates the description of examined variables and their group difference. To reduce variability in variables with larger values, such as information strain, health-related strain, conventional beliefs, internal resilience and conventional beliefs, we took the log transformations of them (Feng et al., 2014). As expected, gender differences were observed in the use of social media (*p* < 0.01). Females spent significantly more time on social media during the pandemic. No significant gender differences were found for pandemic-related strains. Furthermore, females possessed higher levels of conventional beliefs (*p* < 0.001) than males.

Table 2 shows the correlations among the analytical variables for the entire sample. Health-related strain was significantly but negatively related to online gaming. Among three protective variables, internal resilience and life satisfaction showed significant negative associations with online gaming, while only life satisfaction had a significant negative correlation with the use of social media. In addition, internal resilience was significantly and negatively related with economic and family relationship strain. Life satisfaction demonstrated significant negative relationship with all pandemic-related strains, except for health-related strain. Regarding control variables, males were less likely to engage in frequent social media use. Adolescents in Shenzhen had lower levels of economic strain and conventional beliefs. Those who mainly lived with parents had lower levels of economic and family relationship strain, and higher life satisfaction.

### 7.2. Results from the Baseline Model

Table 3 and Table 4 display the results of the baseline model, which predicts the effect of pandemic-related strains on frequent use of games and social media while adjusting for resident city, gender (for the full sample only) and living with parents. For the entire sample, information strain was positively associated with frequent Internet use of games (b = 0.275, *p* < 0.01) and social media (b = 0.190, *p* < 0.05), while health-related strain was negatively associated with these activities (b = −0.331, *p* < 0.001; b = −0.227, *p* < 0.01). This pattern was consistent across gender groups, except for the use of social media for female workers, where no significant association with strains was found. In addition, economic strain had different impacts on two genders. Males with higher economic strains were less likely to play online games (b = −0.235, *p* < 0.1), while females were more likely to do so (b = 0.299, *p* < 0.01).

### 7.3. Moderating Effect

Given that pandemic-related strains significantly affect online gaming among both male and female migrant working adolescents (as reported in Table 3), we conducted a moderation analysis to examine whether three conditioning factors may buffer the relationship between strain and gaming (see Table 5 for male workers and Table 6 for female workers). For social media use, we conducted the moderation analysis only for male workers (see Table 7) since female workers’ social media use is not that sensitive to pandemic-related strains.

**Figure 1:**
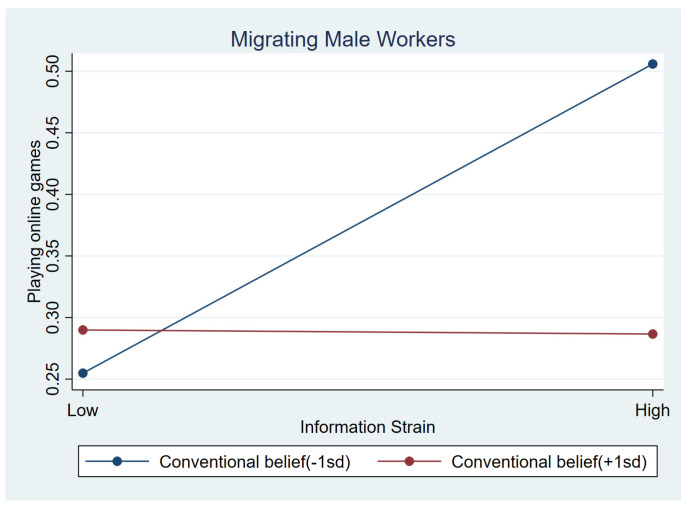
Relationship between information strain and online games at low conventional beliefs (1 SD below the mean) and high conventional beliefs (1 SD above the mean) among male workers.

**Figure 2:**
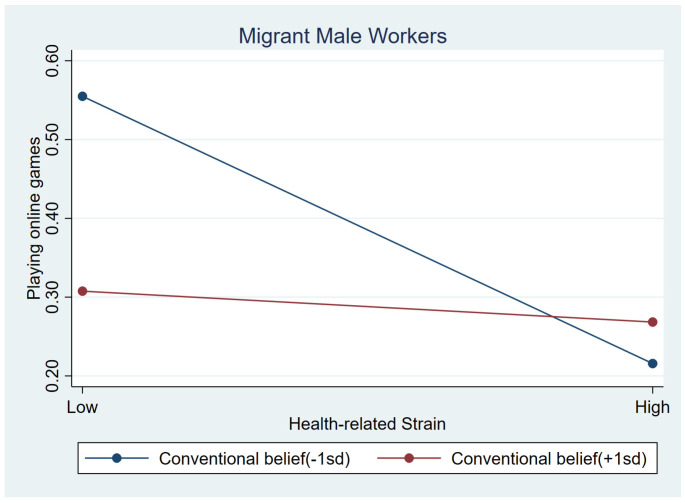
Relationship between health-related strain and online games at low conventional beliefs (1 SD below the mean) and high conventional beliefs (1 SD above the mean) among male workers.

As shown in Table 5, interaction effects showed a slightly significant association between conventional beliefs and two strains, information strain (b = −0.333, *p* < 0.1) and health-related strain (b = 0.362, *p* < 0.1). Simple slope tests indicated that, for migrant male workers with low conventional beliefs (i.e., 1 SD below the mean), those who had higher information strain played online games more frequently (b_simple_ = 0.133, *p* < 0.01), but less frequently when they had stronger conventional beliefs (i.e., 1 SD above the mean), (b_simple_ = −0.002, *p* > 0.1). Figure 1 shows the moderating effect more intuitively. Unexpectedly, those with more health-related strains had lower likelihood for online gaming when they had lower conventional beliefs (b_simple_ = −0.165, *p* < 0.01). This effect was weaker among those with high conventional beliefs (b_simple_ = −0.020, *p* > 0.1). Figure 2 illustrates this association.

**Figure 3:**
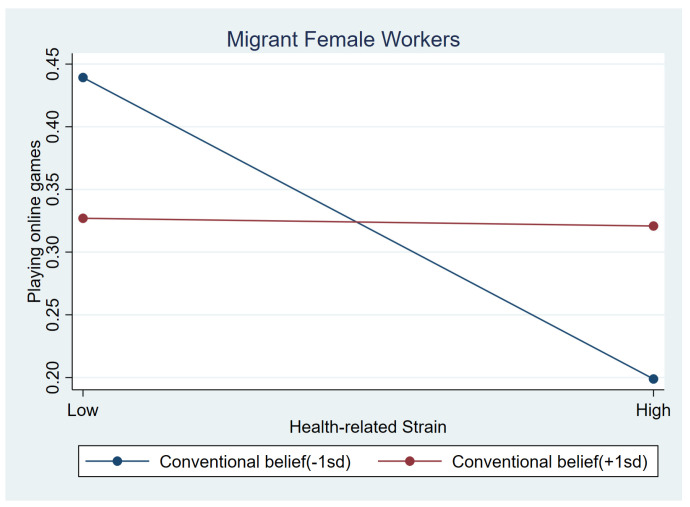
Relationship between health-related strain and online games at low conventional beliefs (1 SD below the mean) and high conventional beliefs (1 SD above the mean) among female workers.

**Figure 4:**
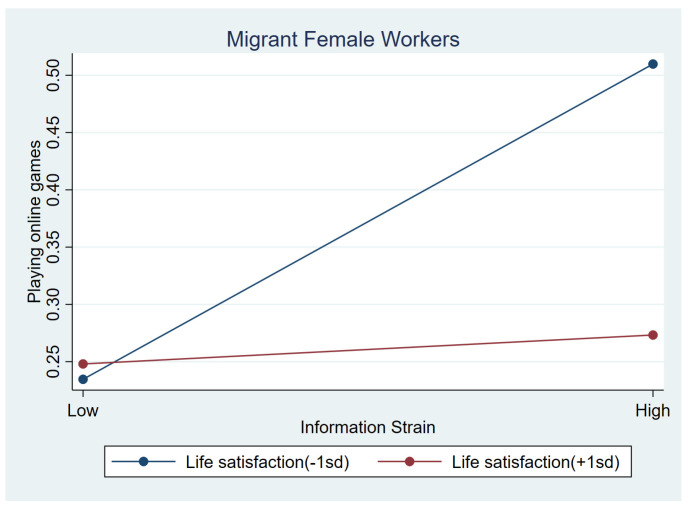
Relationship between information strain and online games at low life satisfaction (1 SD below the mean) and high life satisfaction (1 SD above the mean) among female workers.

**Figure 5:**
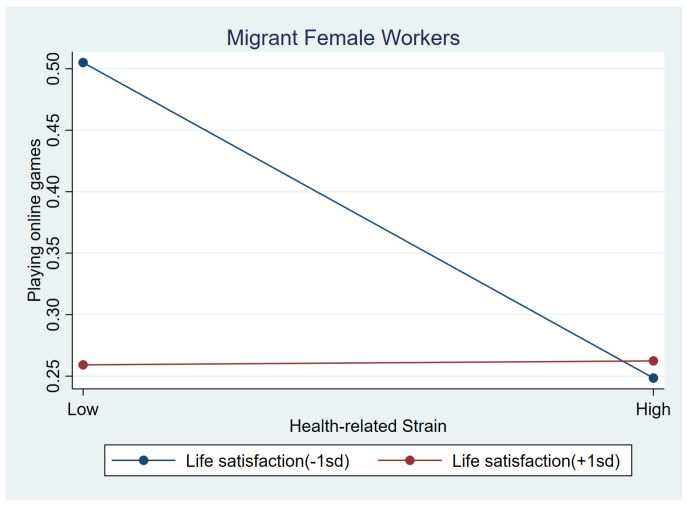
Relationship between health-related strain and online games at low life satisfaction (1 SD below the mean) and high life satisfaction (1 SD above the mean) among female workers.

Table 6 presents the moderating effects of three protective variables on frequent use of online games for females. No significant interaction terms were found for internal resilience. Similar to male workers, among females with low conventional beliefs, the negative association between health-related strain and frequent gaming tend to be stronger than those with higher conventional beliefs (b = 0.302, *p* < 0.1); Figure 3 illustrates this association. Similarly, life satisfaction showed a positive interaction impact with health-related strain (b = 0.311, *p* < 0.05). For information strain, life satisfaction had a negative interaction impact (b = −0.303, *p* < 0.05). Females who experienced more information strain were less likely to play games when they had higher life satisfaction (b_simple_ = 0.013, *p* > 0.1). Figure 4 and Figure 5 illustrate the association between information strain, health-related strain and frequent gaming for female workers with different life satisfaction intuitively.

**Figure 6:**
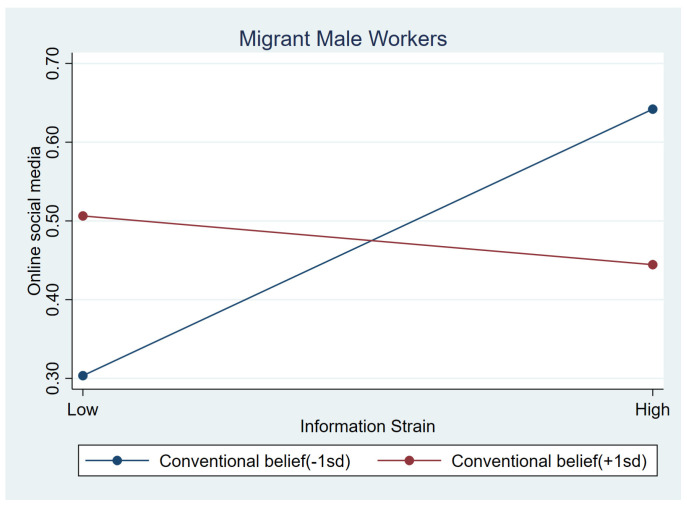
Relationship between information strain and online social media at low conventional beliefs (1 SD below the mean) and high conventional beliefs (1 SD above the mean).

Table 7 demonstrates the moderating effects of three protective variables on frequent use of social media for males. Similar to the moderating impacts on playing games, we only found a significant impact between conventional beliefs and information strain (b = −0.465, *p* < 0.05). It was shown that males use social media less frequently when they reported higher information strain and had stronger conventional beliefs (b_simple_ = −0.031, *p* > 0.1), but more frequently when their conventional beliefs were weaker (b_simple_ = 0.176, *p* < 0.001). This moderating effect is shown in Figure 6.

## 8. Discussion

Stimulated by the strain resulting from the unprecedented Covid-19 pandemic among migrant working adolescents in China, this study concentrates on one of the worst coping strategies, excessive Internet use for gaming, and social media. Based on GST, our study examined the variations between males and females in pandemic-related strains, frequent use of the Internet and the associations among these variables, as well as the mitigating effects of three protective factors on these relationships.

### 8.1. Variations by Gender

First of all, we found that female migrant working adolescents tend to use social media more frequently. This result is in line with expectations and is consistent with previous studies (Noguti et al., 2019). Women are more likely to use social media to maintain social connections and seek emotional support, especially when other offline social interactions were restricted during the pandemic. However, no significant gender differences were found in the use of online games. This trend was also confirmed by another study on Spanish youngsters (Rodriguez-Barcenilla & Ortega-Mohedano, 2022). One possible reason is that the development of games is managing to minimize gender gaps. The increasing presence of female protagonists and narratives in online games highlights the gaming industry’s gradual diversification and its improvement of inclusivity (Ngoc, 2024). In addition, it suggests that when offline leisure activities are restricted, online gaming can be an essential resource for both genders. Accordingly, focusing on young males’ online gaming behavior is no longer sufficient for generalizability. Furthermore, future research could explore additional use patterns, such as consumption habits and motivations, across genders and broader demographic groups. This would complement the current study, which focused only on gaming frequency for a vulnerable group. Motivations are key to understanding use and effect processes, as they can moderate them (Hay et al., 2018). A detailed exploration of gaming behaviors could provide valuable insights into UGT and gender-specific differences in gaming.

Moreover, no significant gender difference was found in pandemic-related strains, suggesting that men and women experienced comparable levels of stress when facing challenges caused by the pandemic. Regarding three protective factors, females tend to have higher levels of conventional beliefs. This finding supports that women have a stronger bond with the society. They are more likely to rely on internalized conventional values to guide behavior and cope with challenges (Rebellon et al., 2016).

### 8.2. Main Effects of Pandemic-Related Strain on the Frequent Use of the Internet

The positive relationship between information strain and frequent Internet use is consistent with our prediction. Being bothered by misinformation can lead to information overload, which is associated with the intention to share information (Ahmed & Rasul, 2022; Bermes, 2021). In other words, migrant working adolescents experiencing information strain may be more likely to use social media to share the pandemic-related information. In addition, information overload is positively associated with mental health problems such as anxiety and depression (J. Wang et al., 2022). For adolescents, online gaming has been an effective way to relieve these negative emotions during pandemic restrictions (Pallavicini et al., 2022). The association between different negative emotions, such as anxiety, depression, and anger, and online gaming can be explored in future research. It would be beneficial to investigate not only the possible existence of such an association but also its nature and direction, contributing to the development of more effective strategies for mental health promotion and behavior modification.

One unexpected finding is the health-related strains are negatively associated with online gaming and social media use for both genders. This implies that health-related strain can inhibit frequent Internet use. A possible explanation is that health-related strains lead to more concerns about activities that could harm health. This motivates people to maintain a healthy lifestyle (Zhu et al., 2021), including engaging in more physical activities, and avoiding unhealthy habits like excessive Internet use. Another study corroborated the positive relationship between the fear of COVID-19 and healthy lifestyle behaviors (Yıldız & Çiftçi, 2023). These results highlight that the concerns on the health consequences of the pandemic can also positively impact certain individuals’ daily behaviors.

Economic strains affect male and female adolescents differently. While economic strain correlates positively with increased online gaming among females, it inhibits online gaming among males. It is possible because many online games favored by males entail a monetary cost. Several studies found that being male was positively linked to spending money on games (Costes & Bonnaire, 2022; Gainsbury et al., 2016). Therefore, as financial strain increases, males are less likely to play games that require monetary investment.

Among adolescent female workers, no significant association was identified between pandemic-related strains and the frequent use of social media. One explanation could be that females tend to use social media frequently regardless of stress level. Their motivation to use social media includes socializing by regularly sharing personal updates and commenting on others’ posts (Hampton et al., 2011). In other words, social media is a platform for females to maintain social connections, a behavior that remains relatively unchanged despite pandemic-related challenges.

### 8.3. The Moderating Effect of Protective Factors

For male migrant working adolescents, conventional beliefs are the only effective moderator for both online activities. Higher conventional beliefs weaken the positive correlation between information strain and frequent Internet use. However, conventional beliefs do not significantly prevent females’ cyber strain coping. This gender difference aligns with some previous offline delinquency studies. For example, one research about Chinese adolescents found that conventional beliefs are more likely to reduce male delinquency than female (Bao et al., 2016). Males with strong conventional beliefs tend to be more committed to the society, which fosters a greater sense of social responsibility and moral restraint, even in times of stress (Hirschi, 1969). Hence, they may be less likely to use social media to share misinformation about the pandemic when they themselves are bothered by information strain. Moreover, these young male workers may try to release their stress through normative means. On the other hand, those with weaker social beliefs might experience more anxiety and depression when facing health-related strains, as they are more concerned about the well-being of themselves instead of the others. This anxiety, regardless of gender, can be negatively related to their Internet use in response to high levels of health-related strain (Thom et al., 2018).

For female migrant working adolescents, life satisfaction is a more important moderator. High life satisfaction can mitigate the facilitating effect of information strain on frequent Internet use. Adolescent female workers with high life satisfaction also consistently demonstrate lower levels of online game playing, regardless of the extent of their health-related strain. This is consistent with previous studies that emphasize the strong protective effect of life satisfaction on women. For example, Beutel et al. (2009) showed that life satisfaction had a significant positive effect on women’s mental health and positive outcomes. The gender difference in the protective impact of life satisfaction in this study may arise from the different life satisfaction domains valued by males and females. Women prioritize relationships such as partner’s happiness, which reduces their need to use online games for happiness. In contrast, men focus on work satisfaction, which is less conflict with improved happiness through the Internet (Milovanska-Farrington & Farrington, 2022). It is crucial to realize that life satisfaction is more preventive for girls than for boys when we design future policies and/or treatments.

### 8.4. Limitations and Future Directions

Although this study yielded many interesting findings, there were some limitations. The first limitation of the present study is its cross-sectional design, which limits the establishment of a strict causal relationship between strains and Internet use. Future studies should consider collecting and analyzing longitudinal data to further clarify the causal relationships. Secondly, the respondents in this study were not broadly representative and only represent a vulnerable group in China. Future research can include adolescents, migrant workers and other groups to compare with the current findings. In areas where culture, education, and containment policies differ from those in China, strains associated with delinquency and the effect of protective factors may also differ. Further studies can be conducted in a broader range of areas to identify regional disparities. Thirdly, our data were based on self-report questionnaires from adolescents. Problems including under-reporting of delinquent behaviors or socially desirable biases can interfere with the accuracy of the results. Further studies can use multiple methods like direct observations to collect a more comprehensive dataset. In addition, the measure of family relationship strain did not include conflicts between children and parents. During the pandemic, conflicts between parents and children have become a concerning issue (Chen et al., 2021). The parent–child relationship is also an essential source of family strain that may impact delinquency. It would be valuable for future studies to examine the effect of parent–child conflicts on delinquent copings. Finally, as the moderating effect was partially supported in this study, future studies can explore such impact of other moderating variables. Positive relationships with friends and positive working climate are worthy of consideration.

### 8.5. Practical Implications

Despite these limitations, our findings have some important practical implications. As females tend to use social media frequently and consistently in daily life, it is important for parents and employers to take targeted interventions. Parents can guide girls to engage in more real-life interactions, such as parties with friends or family members, outdoor activities, and local festivals, to maintain social connections. Employers can organize various offline group activities among staff to encourage more female workers to participate.

Migrant working adolescents, in particular, require more attention regarding information strain, as false information significantly affects their frequent Internet use. Policymakers should strengthen regulations on online information, combat misinformation promptly, and enhance adolescents’ media literacy. Employers hiring young migrant people should prioritize their ability to discern information. In addition, since economic strain can promote online gaming behavior among females, policymakers should create a more female-friendly employment environment and offer more job opportunities for migrate female adolescents. Government assistance and employment support, such as vocational training, can help those facing economic challenges improve their economic stability and vocational skills. Parents should pay more attention to their daughters’ financial situations and provide support whenever possible.

Due to the different impacts of protective factors, the most desirable social policy that prevent the excessive Internet use should cater to migrant working adolescents in different genders. For males, more attention should be paid to their conventional beliefs and cultivate their conventional coping strategies to relieve strains. Male working adolescents should be encouraged to take part in some workshops about discipline and responsibility. Local authorities and employers could also organize and promote volunteering and charities activities to instill a sense of responsibility to the society. For females, emphasis should be placed on enhancing life satisfaction. Employers should ensure reasonable work schedules and provide welfare to assist female working adolescents in maintaining a healthy work-life balance. Providing access to mental health resources and organizing social networking events can further support females’ well-being and satisfaction.

In a broader context, COVID-19 is only one of numerous global threats that cause great damages to our daily lives. Other sudden risk events, such as natural disasters, economic crises, and terrorist attacks, can similarly result in highly disruptive physical, economic, political and social environments. Although these large-scale risks are hard to predict, understanding people’s coping strategies under uncertainty can be beneficial for taking preventive and supportive strategies that mitigate the potential negative impacts. Our findings shed light on stress levels and coping strategies, especially among individuals already facing vulnerabilities, when exposed to such substantial risks. These insights can inform future responses to global crises with more effective measures to support those at-risk populations in coping with such challenges.

## 9. Conclusions

In conclusion, this study provides a comprehensive picture of gendered strain, gendered coping and gendered protectors among migrant working adolescents in China under the pandemic, a large-scale social crisis. This study expands on the offline framework of GST by addressing pandemic-related strains and cyber behavioral coping strategies among migrant working boys and girls in China, a less-studied disadvantaged group with dual vulnerabilities. Additionally, we examined three potential moderators between strain and cyber coping strategies by gender. Our findings revealed partial support for this extended GST model. Notably, we identified that adolescent male workers are more responsive to the protection effects of conventional beliefs, while migrant working girls are more likely to be protected by high level of life satisfaction. These findings have pivotal implications for the development of effective gender sensitive policies to prevent excessive Internet use among migrant working adolescents during sudden risk events and in the long term.

## Figures and Tables

**Table 1:** Descriptive statistics.

		Mean/Percentages (SD)	
	Full (N = 769)	Male (N = 352)	Female (N = 417)
**Digital copings**	0.56 (0.50)	0.51 (0.50)	0.60 * (0.49)
Online games	0.32 (0.47)	0.33 (0.47)	0.31 (0.46)
Social media	0.53 (0.50)	0.48 (0.50)	0.58 ** (0.49)
Pandemic-related Strains			
Economic strain	1.06 (1.12)	1.03 (1.07)	1.09 (1.16)
Information strain	2.20 (0.33)	2.21 (0.36)	2.20 (0.31)
Health-related strain	3.23 (0.43)	3.24 (0.43)	3.23 (0.42)
Family relationship strain	0.17 (0.46)	0.19 (0.50)	0.15 (0.43)
Protective Factors			
Conventional beliefs	3.03 (0.26)	2.98 (0.30)	3.06 *** (0.22)
Internal resilience	1.02 (0.32)	1.01 (0.32)	1.02 (0.32)
Life satisfaction	1.49 (0.28)	1.50 (0.28)	1.48 (0.28)
Control Variables			
Gender			
Male	45.77%		
Female	54.23%		
City			
Shenzhen	58%	65.63%	51.56%
Changsha	42%	34.38%	48.44%
Live with parents			
Yes	40.05%	40.06%	40.05%
No	59.95%	59.94%	59.95%

Note: * *p* < 0.05, ** *p* < 0.01, *** *p* < 0.001; SD, standard deviation; *p*-values derived from chi-square test for dummy variables and independent *t*-test for continuous variables.

**Table 2:** Pearson’s correlations of analytical variables (full sample).

	1	2	3	4	5	6	7	8	9	10	11	12
1 Online game	1											
2 Social media	0.52 ***	1										
3 Economic strain	0.05	0.04	1									
4 Information strain	0.07 ^+^	0.05	0.13 ***	1								
5 Health strain	−0.10 **	−0.07 ^+^	0.11 **	0.42 ***	1							
6 Relationship strain	0.050	0.06	0.25 ***	0.09 *	0.05	1						
7 Conventional beliefs	−0.01	0.03	0.06	−0.002	−0.06 ^+^	0.05	1					
8 Internal resilience	−0.08 *	−0.05	−0.10 **	−0.006	−0.02	−0.11 **	0.03	1				
9 Life satisfaction	−0.08 *	−0.11 **	−0.18 ***	−0.08 *	−0.06 ^+^	−0.14 ***	−0.01	0.12 **	1			
10 Male	0.02	−0.10 **	−0.02	0.01	0.01	0.04	−0.16 ***	−0.01	0.04	1		
11 Shenzhen	−0.02	−0.03	−0.13 ***	0.02	0.10 **	0.07 ^+^	−0.21 ***	−0.01	0.009	0.14 ***	1	
12 Live with parents	−0.03	−0.02	−0.11 **	−0.02	0.043	−0.08 *	0.03	0.03	0.19 ***	0.00	0.08 *	1

Note: ^+^ *p* < 0.10, * *p* < 0.05, ** *p* < 0.01, *** *p* < 0.001.

**Table 3:** Baseline model: logistic regression models predicting playing online games.

**Variables**		**Full Sample** **(N = 769)**				
	**Exp(b)**	**b**			**SE**	
Strain variables						
Economic strain	1.078	0.075			0.083	
Information strain	1.317	0.275 **			0.092	
Health-related strain	0.718	−0.331 ***			0.086	
Family relationship strain	1.079	0.076			0.079	
Control variables						
Male	1.105	0.100			0.159	
Shenzhen	0.948	−0.053			0.164	
Live with parents	0.939	−0.063			0.163	
Nagelkerke’s R^2^		0.039				
**Variables**		**Male** **(N = 352)**			**Female** **(N = 417)**	
	**Exp(b)**	**b**	**SE**	**Exp(b)**	**b**	**SE**
Strain variables						
Economic strain	0.791	−0.235 ^+^	0.134	1.349	0.299 **	0.110
Information strain	1.302	0.264 ^+^	0.138	1.396	0.333 *	0.129
Health-related strain	0.688	−0.373 **	0.132	0.771	−0.260 *	0.117
Family relationship strain	1.143	0.133	0.119	1.048	0.047	0.108
Control variables						
Shenzhen	0.922	−0.081	0.251	0.902	−0.103	0.221
Live with parents	0.807	−0.214	0.243	1.083	0.079	0.224
Nagelkerke’s R^2^		0.058			0.063	

Note: ^+^ *p* < 0.10, * *p* < 0.05, ** *p* < 0.01, *** *p* < 0.001; The statistical significance of coefficients was evaluated through Wald tests.

**Table 4:** Baseline model: logistic regression models predicting online social media.

**Variables**		**Full Sample** **(N = 769)**				
	**Exp(b)**	**b**			**SE**	
Strain variables						
Economic strain	1.042	0.041			0.078	
Information strain	1.210	0.190 *			0.082	
Health-related strain	0.797	−0.227 **			0.083	
Family relationship strain	1.116	0.109			0.078	
Control variables						
Male	0.661	−0.414 **			0.149	
Shenzhen	0.981	−0.019			0.153	
Live with parents	0.948	−0.053			0.151	
Nagelkerke’s R^2^		0.035				
**Variables**		**Male** **(N = 352)**			**Female** **(N = 417)**	
	**Exp(b)**	**b**	**SE**	**Exp(b)**	**b**	**SE**
Strain variables Economic strain	0.961	−0.040	0.119	1.112	0.106	0.105
Information strain	1.283	0.249 *	0.126	1.167	0.154	0.110
Health-related strain	0.724	−0.323 *	0.127	0.879	−0.128	0.111
Family relationship strain	1.117	0.111	0.114	1.117	0.111	0.110
Control variables						
Shenzhen	0.910	−0.094	0.235	1.015	0.015	0.204
Live with parents	0.773	−0.257	0.226	1.137	0.128	0.206
Nagelkerke’s R^2^		0.042			0.018	

Note: * *p* < 0.05, ** *p* < 0.01; the statistical significance of coefficients was evaluated through Wald tests.

**Table 5:** Logistic regression models predicting playing online games for male workers with moderators.

Variables	Model 1		Moderator: Conventional Beliefs		Moderator: Internal Resilience		Moderator: Life Satisfaction	
	b	SE	b	SE	b	SE	b	SE
Strain variables Economic strain	−0.258 ^+^	0.137	−0.249 ^+^	0.136	−0.258 ^+^	0.136	−0.256 ^+^	0.136
Information strain	0.260 ^+^	0.141	0.325 *	0.149	0.302 *	0.149	0.257 ^+^	0.139
Health-related strain	−0.380 **	0.133	−0.458 **	0.146	−0.409 **	0.141	−0.367 **	0.133
Family relationship strain	0.141	0.121	0.150	0.123	0.120	0.123	0.105	0.124
Moderators								
Conventional beliefs	−0.173	0.117	−0.155	0.126				
Internal resilience	−0.077	0.119			−0.076	0.120		
Life satisfaction	−0.094	0.120					−0.086	0.132
M × Economic strain			0.115	0.127	−0.057	0.121	0.082	0.131
M × Information strain			−0.333 ^+^	0.185	−0.011	0.148	−0.103	0.152
M × health-related strain			0.362 ^+^	0.193	0.111	0.148	−0.032	0.156
M × relationship strain			−0.082	0.128	0.130	0.148	−0.077	0.108
Control variables								
Shenzhen	−0.189	0.259	−0.214	0.260	−0.117	0.255	−0.078	0.254
Live with parents	−0.161	0.247	−0.126	0.248	−0.194	0.245	−0.193	0.247
Nagelkerke’s R^2^	0.071		0.088		0.068		0.067	

Note: ^+^ *p* < 0.10, * *p* < 0.05, ** *p* < 0.01; the statistical significance of coefficients was evaluated through Wald tests.

**Table 6:** Logistic regression models predicting playing online games for female workers with moderators.

Variables	Model 1		Moderator: Conventional Beliefs		Moderator: Internal Resilience		Moderator: Life Satisfaction	
	b	SE	b	SE	b	SE	b	SE
Strain variables Economic strain	0.263 *	0.112	0.304 **	0.112	0.283 *	0.113	0.248 *	0.114
Information strain	0.320 *	0.135	0.368 **	0.133	0.422 **	0.149	0.370 **	0.133
Health-related strain	−0.269 *	0.119	−0.317 *	0.125	−0.269 *	0.121	−0.303 *	0.123
Family relationship strain	−0.032	0.114	0.058	0.119	−0.067	0.137	0.050	0.111
Moderators								
Conventional beliefs	0.105	0.125	0.058	0.141				
Internal resilience	−0.233 *	0.112			−0.225 ^+^	0.115		
Life satisfaction	−0.178	0.111					−0.224 ^+^	0.131
M × Economic strain			−0.118	0.127	−0.075	0.108	0.004	0.117
M × Information strain			−0.010	0.128	−0.208	0.155	−0.303 *	0.154
M × health-related strain			0.302 ^+^	0.161	0.153	0.114	0.311 *	0.158
M × relationship strain			−0.048	0.162	−0.061	0.108	0.038	0.110
Control variables								
Shenzhen	−0.033	0.229	−0.057	0.230	−0.082	0.224	−0.056	0.225
Live with parents	0.138	0.231	0.074	0.227	0.064	0.227	0.165	0.230
Nagelkerke’s R^2^	0.090		0.086		0.090		0.094	

Note: ^+^ *p* < 0.10, * *p* < 0.05, ** *p* < 0.01; the statistical significance of coefficients was evaluated through Wald tests.

**Table 7:** Logistic regression models predicting online social media for male workers with moderators.

Variables	Model 1		Moderator: Conventional Beliefs		Moderator: Internal Resilience		Moderator: Life Satisfaction	
	b	SE	b	SE	b	SE	b	SE
Strain variables Economic strain	−0.047	0.120	−0.067	0.121	−0.049	0.120	−0.054	0.120
Information strain	0.246 ^+^	0.128	0.339 *	0.142	0.236 ^+^	0.135	0.253 *	0.129
Health-related strain	−0.323 *	0.127	−0.411 **	0.141	−0.318 *	0.134	−0.339 **	0.130
Family relationship strain	0.110	0.114	0.122	0.116	0.108	0.116	0.080	0.117
Moderators								
Conventional beliefs	−0.03	0.112	0.012	0.122				
Internal resilience	−0.016	0.110			−0.002	0.112		
Life satisfaction	−0.040	0.113					−0.018	0.125
M × Economic strain			0.152	0.118	−0.070	0.109	0.047	0.120
M × Information strain			−0.465 *	0.185	0.061	0.137	−0.195	0.144
M × health-related strain			0.296	0.184	−0.005	0.138	0.126	0.155
M × relationship strain			−0.038	0.122	0.084	0.138	−0.130	0.110
Control variables								
Shenzhen	−0.116	0.241	−0.150	0.242	−0.099	0.238	−0.071	0.238
Live with parents	−0.241	0.228	−0.183	0.230	−0.250	0.227	−0.256	0.230
Nagelkerke’s R^2^	0.043		0.072		0.046		0.057	

Note: ^+^ *p* < 0.10, * *p* < 0.05, ** *p* < 0.01; The statistical significance of coefficients was evaluated through Wald tests.

## Data Availability

The data presented in this study are available on reasonable request from the first author. The data are not publicly available due to ethical considerations.

## References

[B1-behavsci-15-00073] Agnew R. (1992). Foundation for a general strain theory of crime and delinquency. Criminology.

[B2-behavsci-15-00073] Agnew R. (2006). Pressured into crime: An overview of general strain theory.

[B3-behavsci-15-00073] Agnew R. (2013). When criminal coping is likely: An extension of general strain theory. Deviant Behavior.

[B4-behavsci-15-00073] Agnew R., Brezina T., Wright J. P., Cullen F. T. (2002). Strain, personality traits, and delinquency: Extending general strain theory. Criminology.

[B5-behavsci-15-00073] Ahmed S., Rasul M. E. (2022). Social media news use and COVID-19 misinformation engagement: Survey study. Journal of Medical Internet Research.

[B6-behavsci-15-00073] Alheneidi H., AlSumait L., AlSumait D., Smith A. P. (2021). Loneliness and problematic internet use during COVID-19 lock-down. Behavioral Sciences.

[B7-behavsci-15-00073] Ali M. M., Dwyer D. S., Vanner E. A., Lopez A. (2010). Adolescent propensity to engage in health risky behaviors: The role of individual resilience. International Journal of Environmental Research and Public Health.

[B8-behavsci-15-00073] Bao W.-N., Haas A., Xie Y. (2016). Life strain, social control, social learning, and delinquency: The effects of gender, age, and family SES among Chinese adolescents. International Journal of Offender Therapy and Comparative Criminology.

[B9-behavsci-15-00073] Bapuji H., De Bakker F. G., Brown J. A., Higgins C., Rehbein K., Spicer A. (2020). Business and society research in times of the corona crisis.

[B10-behavsci-15-00073] Barua A., Barua A. (2012). Gendering the digital body: Women and computers. AI & Society.

[B11-behavsci-15-00073] Bermes A. (2021). Information overload and fake news sharing: A transactional stress perspective exploring the mitigating role of consumers’ resilience during COVID-19. Journal of Retailing and Consumer Services.

[B12-behavsci-15-00073] Beutel M. E., Glaesmer H., Decker O., Fischbeck S., Brähler E. (2009). Life satisfaction, distress, and resiliency across the life span of women. Menopause.

[B13-behavsci-15-00073] Block J., Kremen A. M. (1996). IQ and ego-resiliency: Conceptual and empirical connections and separateness. Journal of Personality and Social Psychology.

[B14-behavsci-15-00073] Borg I., Hermann D. (2023). Attitudes toward crime (s) and their relations to gender, age, and personal values. Current Research in Behavioral Sciences.

[B15-behavsci-15-00073] Brodeur A., Gray D., Islam A., Bhuiyan S. (2021). A literature review of the economics of COVID-19. Journal of Economic Surveys.

[B16-behavsci-15-00073] Broidy L., Agnew R. (1997). Gender and crime: A general strain theory perspective. Journal of Research in Crime and Delinquency.

[B17-behavsci-15-00073] Cai J., Guo W. (2020). Review and prospects of 40 years of market-oriented reform in factor allocation in shenzhen.

[B18-behavsci-15-00073] Campbell-Sills L., Stein M. B. (2007). Psychometric analysis and refinement of the connor–davidson resilience scale (CD-RISC): Validation of a 10-item measure of resilience. Journal of Traumatic Stress: Official Publication of The International Society for Traumatic Stress Studies.

[B19-behavsci-15-00073] Cao C., Wang L., Fang R., Liu P., Bi Y., Luo S., Grace E., Olff M. (2022). Anxiety, depression, and PTSD symptoms among high school students in china in response to the COVID-19 pandemic and lockdown. Journal of Affective Disorders.

[B20-behavsci-15-00073] Chan K. W. (2010). The household registration system and migrant labor in China: Notes on a debate. Population and Development Review.

[B21-behavsci-15-00073] Chang F.-C., Miao N.-F., Chiu C.-H., Chen P.-H., Lee C.-M., Chiang J.-T., Chuang H.-Y. (2016). Urban–rural differences in parental Internet mediation and adolescents’ Internet risks in Taiwan. Health, Risk & Society.

[B22-behavsci-15-00073] Chapple C. L., McQuillan J. A., Berdahl T. A. (2005). Gender, social bonds, and delinquency: A comparison of boys’ and girls’ models. Social Science Research.

[B23-behavsci-15-00073] Chen Y.-X., Cui L.-R., Liu L., Lu F.-R. (2021). Parent-child conflict among primary and middle school students during the COVID-19 epidemic and its countermeasures. Chinese Journal of School Health.

[B24-behavsci-15-00073] Costes J.-M., Bonnaire C. (2022). Spending money in free-to-play games: Sociodemographic characteristics, motives, impulsivity and Internet gaming disorder specificities. International Journal of Environmental Research and Public Health.

[B25-behavsci-15-00073] Dhir A., Chen S., Nieminen M. (2015). A repeat cross-sectional analysis of the psychometric properties of the Compulsive Internet Use Scale (CIUS) with adolescents from public and private schools. Computers & Education.

[B26-behavsci-15-00073] Du J., Mayer G., Hummel S., Oetjen N., Gronewold N., Zafar A., Schultz J.-H. (2020). Mental health burden in different professions during the final stage of the COVID-19 lockdown in China: Cross-sectional survey study. Journal of Medical Internet Research.

[B27-behavsci-15-00073] Duan L., Shao X., Wang Y., Huang Y., Miao J., Yang X., Zhu G. (2020). An investigation of mental health status of children and adolescents in china during the outbreak of COVID-19. Journal of Affective Disorders.

[B28-behavsci-15-00073] Dufour M., Brunelle N., Tremblay J., Leclerc D., Cousineau M.-M., Khazaal Y., Légaré A.-A., Rousseau M., Berbiche D. (2016). Gender difference in internet use and internet problems among Quebec high school students. The Canadian Journal of Psychiatry.

[B29-behavsci-15-00073] Elmer T., Mepham K., Stadtfeld C. (2020). Students under lockdown: Comparisons of students’ social networks and mental health before and during the COVID-19 crisis in Switzerland. PLoS ONE.

[B30-behavsci-15-00073] Erdogan E., Ozdogan O., Erdogan M. (2015). University students’ resilience level: The effect of gender and faculty. Procedia-Social and Behavioral Sciences.

[B31-behavsci-15-00073] Eysenbach G. (2002). Infodemiology: The epidemiology of (mis) information. The American Journal of Medicine.

[B32-behavsci-15-00073] Feng C., Wang H., Lu N., Chen T., He H., Lu Y., Tu X. M. (2014). Log-transformation and its implications for data analysis. Shanghai Archives of Psychiatry.

[B33-behavsci-15-00073] Fergus S., Zimmerman M. A. (2005). Adolescent resilience: A framework for understanding healthy development in the face of risk. Annu. Rev. Public Health.

[B34-behavsci-15-00073] Fernández-Pacheco Alises G., Torres-Jiménez M., Martins P. C., Mendes S. M. V. (2022). Analysing the relationship between immigrant status and the severity of offending behaviour in terms of individual and contextual factors. Frontiers in Psychology.

[B35-behavsci-15-00073] Fredrickson B. L., Levenson R. W. (1998). Positive emotions speed recovery from the cardiovascular sequelae of negative emotions. Cognition & Emotion.

[B36-behavsci-15-00073] Friedman J., Rosenbaum D. P. (1988). Social control theory: The salience of components by age, gender, and type of crime. Journal of Quantitative Criminology.

[B37-behavsci-15-00073] Gadermann A. C., Thomson K. C., Richardson C. G., Gagné M., McAuliffe C., Hirani S., Jenkins E. (2021). Examining the impacts of the COVID-19 pandemic on family mental health in Canada: Findings from a national cross-sectional study. BMJ Open.

[B38-behavsci-15-00073] Gainsbury S. M., King D. L., Russell A. M., Delfabbro P. (2016). Who pays to play freemium games? The profiles and motivations of players who make purchases within social casino games. Journal of Behavioral Addictions.

[B39-behavsci-15-00073] Gao J., Zheng P., Jia Y., Chen H., Mao Y., Chen S., Wang Y., Fu H., Dai J. (2020). Mental health problems and social media exposure during COVID-19 outbreak. PLoS ONE.

[B40-behavsci-15-00073] Gao Y., Wong D. S. (2018). Strains and delinquency of migrant adolescents in China: An investigation from the perspective of general strain theory. Youth & Society.

[B41-behavsci-15-00073] Gilligan C. (1993). In a different voice: Psychological theory and women’s development.

[B42-behavsci-15-00073] Giudice V., Iannaccone T., Faiella F., Ferrara F., Aversano G., Coppola S., De Chiara E., Romano M. G., Conti V., Filippelli A. (2022). Gender differences in the impact of COVID-19 pandemic on mental health of Italian academic workers. Journal of Personalized Medicine.

[B43-behavsci-15-00073] Gloster A. T., Lamnisos D., Lubenko J., Presti G., Squatrito V., Constantinou M., Nicolaou C., Papacostas S., Aydın G., Chong Y. Y. (2020). Impact of COVID-19 pandemic on mental health: An international study. PLoS ONE.

[B44-behavsci-15-00073] Gong Z., Wang L., Wang H. (2021). Perceived stress and internet addiction among Chinese college students: Mediating effect of procrastination and moderating effect of flow. Frontiers in Psychology.

[B45-behavsci-15-00073] (2023). Government of Yuhua District, Changsha, Population. http://www.yuhua.gov.cn/mlyh/rwls/202303/t20230330_11045714.html#:~:text=%E5%B9%B4%E6%9C%AB%E5%85%A8%E5%8C%BA%E5%B8%B8%E4%BD%8F%E4%BA%BA%E5%8F%A3,%E5%9F%8E%E9%95%87%E5%8C%96%E7%8E%87%E4%B8%BA97.5%25%E3%80%82.

[B46-behavsci-15-00073] Hampton K. N., Goulet L. S., Rainie L., Purcell K. (2011). Social networking sites and our lives.

[B47-behavsci-15-00073] Hay J., Grossberg L., Wartella E., Hay J. (2018). The audience and its landscape.

[B48-behavsci-15-00073] He A. J., Zhang C., Qian J. (2022). COVID-19 and social inequality in China: The local–migrant divide and the limits of social protections in a pandemic. Policy and Society.

[B49-behavsci-15-00073] Hidalgo-Fuentes S., Martí-Vilar M., Ruiz-Ordoñez Y. (2023). Problematic internet use and resilience: A systematic review and meta-analysis. Nursing Reports.

[B50-behavsci-15-00073] Hill J. P., Lynch M. E. (1983). The intensification of gender-related role expectations during early adolescence. Girls at puberty: Biological and psychosocial perspectives.

[B51-behavsci-15-00073] Hirschi T. (1969). Causes of delinquency.

[B52-behavsci-15-00073] Hossain M. (2021). Gender differences in experiencing coronavirus-triggered economic hardship: Evidence from four developing countries. Research in Social Stratification and Mobility.

[B53-behavsci-15-00073] Hou F., Bi F., Jiao R., Luo D., Song K. (2020). Gender differences of depression and anxiety among social media users during the COVID-19 outbreak in China: A cross-sectional study. BMC Public Health.

[B54-behavsci-15-00073] Inchley J., Currie D., Young T., Samdal O., Torsheim T., Augustson L. (2016). Growing up unequal: Gender and socioeconomic differences in young people’s health and well-being. Health Behaviour in School-aged Children (HBSC) study: International report from the 2013/2014 survey.

[B55-behavsci-15-00073] Jiang S. (2020). Infodemic: Study on the spread of and response to rumors about COVID-19. Sudies on Science Popularization.

[B56-behavsci-15-00073] Jillani U., Bhutto Z. H., Ahmad K. B. (2023). Emotional Intelligence, Resilience And University Adjustment Of Students: Gender Based Comparative Study. Journal of Positive School Psychology.

[B57-behavsci-15-00073] Junger-Tas J. (1992). An empirical test of social control theory. Journal of Quantitative Criminology.

[B58-behavsci-15-00073] Katz E. (1974). Utilization of mass communication by the individual. The uses of mass communications: Current perspectives on gratifications research.

[B59-behavsci-15-00073] Katz E., Blumler J. G., Gurevitch M. (1973). Uses and gratifications research. The Public Opinion Quarterly.

[B60-behavsci-15-00073] Kim J., LaRose R., Peng W. (2009). Loneliness as the cause and the effect of problematic Internet use: The relationship between Internet use and psychological well-being. Cyberpsychology & Behavior.

[B61-behavsci-15-00073] Kim J., Lee D., Chung Y. (2013). The impact of academic stress on delinquent behavior-Focusing on the mediating effect of depression. Journal of the Korean Society of Child Welfare.

[B62-behavsci-15-00073] Kobayasi R., Tempski P. Z., Arantes-Costa F. M., Martins M. A. (2018). Gender differences in the perception of quality of life during internal medicine training: A qualitative and quantitative analysis. BMC Medical Education.

[B63-behavsci-15-00073] Leonhardt M., Overå S. (2021). Are there differences in video gaming and use of social media among boys and girls?—A mixed methods approach. International Journal of Environmental Research and Public Health.

[B64-behavsci-15-00073] Li L., Liu L., Niu Z., Zhong H., Mei S., Griffiths M. D. (2023). Gender differences and left-behind experiences in the relationship between gaming disorder, rumination and sleep quality among a sample of Chinese university students during the late stage of the COVID-19 pandemic. Frontiers in Psychiatry.

[B65-behavsci-15-00073] Li P., Li W. (2007). Economic status and social attitudes of migrant workers in China. China & World Economy.

[B66-behavsci-15-00073] Li P., Zhuo Q. (2023). Emotional straying: Flux and management of women’s emotions in social media. PLoS ONE.

[B67-behavsci-15-00073] Lin M.-P. (2020). Prevalence of internet addiction during the COVID-19 outbreak and its risk factors among junior high school students in Taiwan. International Journal of Environmental Research and Public Health.

[B68-behavsci-15-00073] Liu Q.-Q., Zhang D.-J., Yang X.-J., Zhang C.-Y., Fan C.-Y., Zhou Z.-K. (2018). Perceived stress and mobile phone addiction in Chinese adolescents: A moderated mediation model. Computers in Human Behavior.

[B69-behavsci-15-00073] Lo T. W., Jiang G. (2006). Inequality, crime and the floating population in China. Asian Journal of Criminology.

[B70-behavsci-15-00073] Lu D., Lai C. (2009). Viewing policy adjustments and changes of “control dropout” in China from student voluntary dropout. Education Research Monthly 2009.

[B71-behavsci-15-00073] Martin K., Huebner E. S., Valois R. F. (2008). Does life satisfaction predict victimization experiences in adolescence?. Psychology in the Schools.

[B72-behavsci-15-00073] Mazerolle P., Maahs J. (2000). General strain and delinquency: An alternative examination of conditioning influences. Justice Quarterly.

[B73-behavsci-15-00073] Merton R. (1938). Social structure and Anomie. American Sociological Review.

[B74-behavsci-15-00073] Metin A., Erbiçer E. S., Şen S., Çetinkaya A. (2022). Gender and COVID-19 related fear and anxiety: A meta-analysis. Journal of Affective Disorders.

[B75-behavsci-15-00073] Milovanska-Farrington S., Farrington S. (2022). Happiness, domains of life satisfaction, perceptions, and valuation differences across genders. Acta Psychologica.

[B76-behavsci-15-00073] Newman G. R., Wolfgang M. E. (2017). Comparative deviance: Perception and law in six cultures.

[B77-behavsci-15-00073] Newman K. L., Jeve Y., Majumder P. (2022). Experiences and emotional strain of NHS frontline workers during the peak of the COVID-19 pandemic. International Journal of Social Psychiatry.

[B78-behavsci-15-00073] Ngoc M. T. L. (2024). Spotlighting women gamers and how they play and spend on video games.

[B79-behavsci-15-00073] Noguti V., Singh S., Waller D. S. (2019). Gender differences in motivations to use social networking sites. Gender economics: Breakthroughs in research and practice.

[B80-behavsci-15-00073] Nsoesie E. O., Oladeji O. (2020). Identifying patterns to prevent the spread of misinformation during epidemics. The Harvard Kennedy School Misinformation Review.

[B81-behavsci-15-00073] Pallavicini F., Pepe A., Mantovani F. (2022). The effects of playing video games on stress, anxiety, depression, loneliness, and gaming disorder during the early stages of the COVID-19 pandemic: PRISMA systematic review. Cyberpsychology, Behavior, and Social Networking.

[B82-behavsci-15-00073] Pearlin L. I., Bierman A. (2013). Current issues and future directions in research into the stress process. Handbook of the Sociology of Mental Health.

[B83-behavsci-15-00073] Rauma D. (1991). The context of normative consensus: An expansion of the Rossi/Berk consensus model, with an application to crime seriousness. Social Science Research.

[B84-behavsci-15-00073] Rebellon C. J., Manasse M. E., Agnew R., Van Gundy K. T., Cohn E. S. (2016). The relationship between gender and delinquency: Assessing the mediating role of anticipated guilt. Journal of Criminal Justice.

[B85-behavsci-15-00073] Rodriguez-Barcenilla E., Ortega-Mohedano F. (2022). Moving towards the end of gender differences in the habits of use and consumption of mobile video games. Information.

[B86-behavsci-15-00073] Schnettler B., Miranda-Zapata E., Orellana L., Saracostti M., Poblete H., Lobos G., Adasme-Berríos C., Lapo M., Beroiza K., Concha-Salgado A. (2024). Intra-and Inter-Individual Associations of Family-to-Work Conflict, Psychological Distress, and Job Satisfaction: Gender Differences in Dual-Earner Parents during the COVID-19 Pandemic. Behavioral Sciences.

[B87-behavsci-15-00073] Sebastião R., Neto D. D., Costa V. (2023). Understanding Differential Stress and Mental Health Reactions to COVID-19-Related Events. International Journal of Environmental Research and Public Health.

[B88-behavsci-15-00073] Sherry J. L., Greenberg B. S., Lucas K., Lachlan K. (2012). Video game uses and gratifications as predictors of use and game preference. Playing video games.

[B89-behavsci-15-00073] Socal M. P., Sharfstein J. M., Greene J. A. (2021). The pandemic and the supply chain: Gaps in pharmaceutical production and distribution.

[B90-behavsci-15-00073] (2002). State Council of the People’s Republic of China. http://www.moe.gov.cn/jyb_xxgk/moe_1777/moe_1778/tnull_27719.html.

[B91-behavsci-15-00073] Steinberg L. (2005). Cognitive and affective development in adolescence. Trends in Cognitive Sciences.

[B92-behavsci-15-00073] Stevens M. W., Dorstyn D., Delfabbro P. H., King D. L. (2021). Global prevalence of gaming disorder: A systematic review and meta-analysis. Australian & New Zealand Journal of Psychiatry.

[B93-behavsci-15-00073] Styck K. M., Malecki C. K., Ogg J., Demaray M. K. (2021). Measuring COVID-19-related stress among 4th through 12th grade students. School Psychology Review.

[B94-behavsci-15-00073] Suldo S. M., Huebner E. S. (2004). The role of life satisfaction in the relationship between authoritative parenting dimensions and adolescent problem behavior. Social Indicators Research.

[B95-behavsci-15-00073] Tang S., Li X. (2021). Responding to the pandemic as a family unit: Social impacts of COVID-19 on rural migrants in China and their coping strategies. Humanities and Social Sciences Communications.

[B96-behavsci-15-00073] Taylor S., Landry C. A., Paluszek M. M., Fergus T. A., McKay D., Asmundson G. J. (2020). Development and initial validation of the COVID Stress Scales. Journal of Anxiety Disorders.

[B97-behavsci-15-00073] Thom R. P., Bickham D. S., Rich M. (2018). Internet use, depression, and anxiety in a healthy adolescent population: Prospective cohort study. JMIR Mental Health.

[B98-behavsci-15-00073] Tu W., Nie Y., Liu Q. (2023). Does the effect of stress on smartphone addiction vary depending on the gender and type of addiction?. Behavioral Sciences.

[B99-behavsci-15-00073] Veenhoven R. (2017). Measures of happiness: Which to choose?. Metrics of subjective well-being: Limits and improvements.

[B100-behavsci-15-00073] Villaume S. C., Stephens J. E., Nwafor E. E., Umaña-Taylor A. J., Adam E. K. (2021). High parental education protects against changes in adolescent stress and mood early in the COVID-19 pandemic. Journal of Adolescent Health.

[B101-behavsci-15-00073] Waller N. G., Lilienfeld S. O., Tellegen A., Lykken D. T. (1991). The tridimensional personality questionnaire: Structural validity and comparison with the multidimensional personality questionnaire. Multivariate Behavioral Research.

[B102-behavsci-15-00073] Wang C., Pan R., Wan X., Tan Y., Xu L., Ho C. S., Ho R. C. (2020). Immediate psychological responses and associated factors during the initial stage of the 2019 coronavirus disease (COVID-19) epidemic among the general population in China. International Journal of Environmental Research and Public Health.

[B103-behavsci-15-00073] Wang J., Huang X., Wang Y., Wang M., Xu J., Li X. (2022). COVID-19 information overload, negative emotions and posttraumatic stress disorder: A cross-sectional study. Frontiers in Psychiatry.

[B104-behavsci-15-00073] Wang X., Zhang J., Wang X., Liu J. (2020). Intervening paths from strain to delinquency among high school and vocational school students in China. International Journal of Offender Therapy and Comparative Criminology.

[B105-behavsci-15-00073] Wegmann E., Brandtner A., Brand M. (2021). Perceived strain due to COVID-19-related restrictions mediates the effect of social needs and fear of missing out on the risk of a problematic use of social networks. Frontiers in Psychiatry.

[B106-behavsci-15-00073] Wiatrowski M. D., Swatko M. K. (1979). Social control theory and delinquency: A multivariate test.

[B107-behavsci-15-00073] Witteveen D. (2020). Sociodemographic inequality in exposure to COVID-19-induced economic hardship in the United Kingdom. Research in Social Stratification and Mobility.

[B108-behavsci-15-00073] Wolfgang M. E. (1985). The national survey of crime severity.

[B109-behavsci-15-00073] Wong D. F. K., Li C. Y., Song H. X. (2007). Rural migrant workers in urban China: Living a marginalised life. International Journal of Social Welfare.

[B110-behavsci-15-00073] Wu Q., Ge T., Emond A., Foster K., Gatt J. M., Hadfield K., Mason-Jones A. J., Reid S., Theron L., Ungar M. (2018). Acculturation, resilience, and the mental health of migrant youth: A cross-country comparative study. Public Health.

[B111-behavsci-15-00073] Wu Q., Ren Q., Zhong N., Bao J., Zhao Y., Du J., Chen T., Zhao M. (2022). Internet behavior patterns of adolescents before, during, and after COVID-19 pandemic. Frontiers in Psychiatry.

[B112-behavsci-15-00073] Wu Q., Tsang B., Ming H. (2014). Social capital, family support, resilience and educational outcomes of Chinese migrant children. British Journal of Social Work.

[B113-behavsci-15-00073] Wu S., Zhang K., Parks-Stamm E. J., Hu Z., Ji Y., Cui X. (2021). Increases in anxiety and depression during COVID-19: A large longitudinal study from China. Frontiers in Psychology.

[B114-behavsci-15-00073] Yıldız E., Çiftçi M. Ç. (2023). The relationship between COVID-19 fear levels and healthy lifestyle behaviors of elderly individuals: A cross-sectional study. Psychogeriatrics.

[B115-behavsci-15-00073] Zhou S.-J., Zhang L.-G., Wang L.-L., Guo Z.-C., Wang J.-Q., Chen J.-C., Liu M., Chen X., Chen J.-X. (2020). Prevalence and socio-demographic correlates of psychological health problems in Chinese adolescents during the outbreak of COVID-19. European Child & Adolescent Psychiatry.

[B116-behavsci-15-00073] Zhu S., Zhuang Y., Ip P. (2021). Impacts on children and adolescents’ lifestyle, social support and their association with negative impacts of the COVID-19 pandemic. International Journal of Environmental Research and Public Health.

